# A *C. elegans* model of copper deficiency: Dietary interventions rescue CTR1/CHCA-1 copper transporter mutant phenotype

**DOI:** 10.1371/journal.pgen.1012013

**Published:** 2026-01-23

**Authors:** Yang Fu, Xu Bai, Lei Chun, X. Z. Shawn Xu, Jianfeng Liu

**Affiliations:** 1 College of Life Science and Technology, Key Laboratory of Molecular Biophysics of MOE, Huazhong University of Science and Technology, Wuhan, Hubei, China; 2 Life Sciences Institute and Department of Molecular and Integrative Physiology, University of Michigan, Ann Arbor, Michigan, United States of America; University of Miami, UNITED STATES OF AMERICA

## Abstract

Copper is an essential micronutrient for all living organisms. Mutations in the copper-importing transporter CTR1/CHCA-1 are associated with a severe copper deficiency disorder in humans, for which no effective cures are currently available. Here, we develop *C. elegans* as a model for copper deficiency. We show that *chca-1* mutant worms fed HT115 bacterial diet exhibited a severe developmental phenotype resulting from copper deficiency, reminiscent of the symptoms observed in human patients. Remarkably, this phenotype can be rescued by switching to OP50 bacterial diet or by supplementing HT115 bacterial diet with glutathione disulfide (GSSG), a metabolite enriched in OP50. Such dietary interventions remodeled the transcriptome of *chca-1* mutants towards that of wild-type worms and upregulated the expression of CTR1/CHCA-1-like copper transporters, thereby ameliorating the mutant phenotype. Our findings establish *C. elegans* as a model for copper deficiency caused by CTR1/CHCA-1, suggesting that dietary interventions may offer a potential therapeutic approach for this severe disease.

## Author summary

Copper is an essential micronutrient for all living organisms. Mutations in the copper transporter CTR1 are associated with severe copper deficiency disorders in humans, for which no effective therapies currently exist. In this study, we established the nematode *C. elegans* as a model for copper deficiency. We found that worms with mutations in *chca-1*, the worm ortholog of human CTR1, exhibit severe developmental delay when fed HT115 bacteria, a phenotype resulting from copper deficiency. This developmental defect can be rescued by either switching the bacterial diet to OP50 or by supplementing HT115 with glutathione disulfide (GSSG), a metabolite naturally enriched in OP50 bacteria. These dietary interventions restore normal gene expression patterns and upregulate compensatory copper transporters in the mutant worms. Our findings identify dietary interventions as potential therapeutic strategies for copper deficiency disorders caused by defects in copper transport.

## Introduction

Copper is a cofactor of many vital enzymes, such as cytochrome C oxidase, Cu/Zn superoxide dismutases, dopamine β-hydroxylase, lysyl oxidase, and copper amine oxidases, which mediate multiple cellular processes, ranging from mitochondrial energy production to free radical detoxification and iron mobilization [1–3]. As such, copper is an essential micronutrient for all living organisms. Copper is transported into the cell mainly via the high-affinity copper-importing transporter CTR1 (encoded by the *SLC31A1* gene in mammals) [4,5]. In addition, copper can be exported out of the cell via two copper-exporting ATPases, ATP7A and ATP7B [6–8]. Notably, mutations in the copper-importing (uptake) transporter CTR1 in humans cause a severe copper deficiency disorder characterized by profound developmental deficits such as global developmental delay, multisystem anomalies, rapid brain atrophy and early death, for which no effective cures are currently available [9,10]. Therefore, it is imperative to develop therapeutics for this severe disease. However, the lack of animal models for copper deficiency impedes such an effort.

*C. elegans* serves as a valuable genetic model for various human diseases, such as neurodegenerative disorders [11–15], polycystic kidney disease [16–18], diabetes [19], obesity [20,21], and cancer [22]. Compared with traditional animal models like rodents and primates, *C. elegans* features a short generation time and lifespan, small body size, large brood size, completely sequenced genome, and powerful genetic tools, as well as conserved mechanisms that underlie most, if not all, cellular processes [23]. The *C. elegans* CHCA-1 has been identified as the ortholog of human CTR1 [24,25]. However, no *C. elegans* model for CTR1-dependent copper deficiency disorder is available, as mutant worms lacking CTR1/CHCA-1 display no obvious phenotype when fed OP50 bacteria unless dietary copper concentration is artificially reduced to very low levels using copper chelators [25,26]. On the other hand, CTR1 knockout mice are embryonic lethal, offering limited information [4,27].

Here, we developed a *C. elegans* model for copper deficiency. We found that *chca-1* null mutant worms fed *E. coli* HT115, a commonly used bacterial diet for *C. elegans*, exhibited a severe copper deficiency and developmental delay phenotype, reminiscent of that observed in human patients. A *chca-1* allele, engineered by CRISPR to carry a L63P point mutation found in human patients, manifested a similar phenotype. Surprisingly, switching the diet to *E. coli* OP50, another commonly used bacterial diet, led to a strong rescue effect on the copper deficiency and developmental delay phenotype of *chca-1* mutant worms. Supplementing HT115 diet with a small amount of OP50 bacteria or glutathione disulfide (GSSG), a metabolite enriched in OP50 bacteria, also ameliorated the *chca-1* mutant phenotype. Such dietary interventions remodeled the transcriptome of *chca-1* mutant worms towards that of wild-type worms and upregulated the expression of CTR1/CHCA-1-like copper transporters, thereby ameliorating the mutant phenotype. These results not only develop *C. elegans* as an animal model for CTR1/CHCA-1-dependent copper deficiency, but also uncover dietary interventions as a potential therapeutic strategy for this disorder.

## Results

### The copper-importing transporter CHCA-1 is essential for *C. elegans* development when fed on *E. coli* HT115

**Fig 1:**
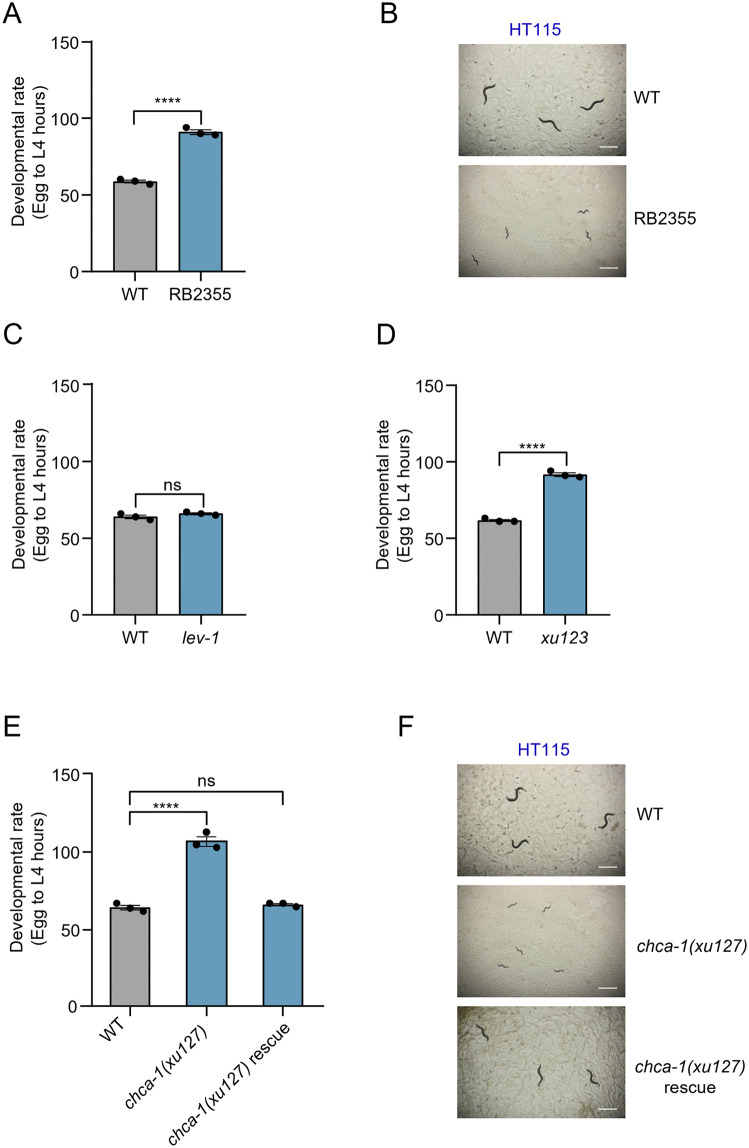
*chca-1* gene is essential for the development of *C. elegans* fed on HT115 diet. **(A)** RB2355 worms manifest a severe developmental delay phenotype when fed HT115. RB2355 is the strain name of *lev-1(ok3201)* from the Caenorhabditis Genetics Center (CGC). ****: P < 0.0001 (t-test). **(B)** Representative images of wild-type and RB2355 worms when fed HT115. The images were taken at 96 hours following seeding synchronized eggs on NGM plates. Scale bar: 1 mm. **(C, D)** *lev-1(xu100)* deletion mutant worms generated by CRISPR (**C**) exhibit a normal developmental rate, while *chca-1(xu123)* mutant (**D**) displays a severe developmental delay compared with wild-type when fed HT115. ns indicates no significant difference, ****: P < 0.0001 (t-test). **(E)** *chca-1(xu127)* mutant worms generated by CRISPR manifest a severe developmental delay when fed on HT115, a phenotype that can be rescued by transgenic expression of *chca-1* wild-type gene using its own promoter. ns: no significant difference, ****: P < 0.0001 (one-way ANOVA with Dunnett’s test). **(F)** Representative images of wild-type, *chca-1(xu127)*, and *chca-1(xu127)* carrying a rescue transgene. All strains were fed on HT115. The images were taken at 96 hours following seeding synchronized eggs on NGM plates. Scale bar: 1 mm. The developmental assay was performed at 20°C in three independent biological replicates (N = 3, n > 50). The hours needed for eggs to develop into mid L4 larvae were scored. Data are presented as the mean ± s.e.m.

HT115 and OP50 are two *E. coli* bacteria strains commonly used to feed *C. elegans* in the laboratory [28,29]. As HT115 and OP50 represent two distinct types of diet with the former being a K-12 strain and the latter a B strain, they are also used to study the effect of diet on various biological processes [30–34]. Nicotinic acetylcholine receptors (nAChRs) represent a group of cation channels regulating neuronal signaling and development [35]. In search for nAChR genes that may function in a diet-dependent manner, we screened mutants lacking nAChRs and found that the strain RB2355, which carries a deletion in the nAChR gene *lev-1,* manifested a severe developmental delay phenotype when fed HT115 but not OP50 (Fig 1A and 1B and see below). Specifically, when fed on HT115 diet, it took a much longer time for the RB2355 strain to develop from eggs to L4 larvae compared to wild-type (Fig 1A and 1B). To verify this developmental phenotype, we generated additional *lev-1* mutant alleles by CRISPR [36]. Surprisingly, such *lev-1* mutant worms exhibited a normal developmental rate when fed HT115 (Fig 1C). We hypothesized that a mutation in a gene other than *lev-1* in the RB2355 background might have caused the developmental delay phenotype. Therefore, we crossed the *lev-1* mutation out of RB2355 and isolated a *xu123* mutant allele, with severe developmental delay when cultured on HT115 bacteria (Fig 1D). By single nucleotide polymorphism (SNP)-based mapping coupled with whole-genome sequencing (WGS) [37,38], we mapped the mutation to the *chca-1* gene, which encodes a homolog of the human copper-importing transporter CTR1. CTR1 is a high-affinity copper uptake protein responsible for importing copper into the cell, which is essential for mammalian development and copper homeostasis [4,5]. To validate this phenotype, we generated *xu127*, a deletion allele of *chca-1,* which contains a 427 bp deletion, resulting in frameshift and premature stop codon that truncates all of the three transmembrane domains essential for CHCA-1 function [39]. This deletion allele is likely a null allele of *chca-1.* We thus focused on characterizing this *chca-1* null allele in all subsequent experiments, referring to it throughout as the *chca-1* mutant unless otherwise specified. Notably, this *chca-1* mutant, when fed HT115, exhibited a severe developmental delay phenotype similar to that observed in the RB2355 strain (Fig 1E and 1F). Transgenic expression of *chca-1* wild-type gene driven by its own promoter *chca-1* or the intestine-specific promoter *ges-1* rescued the developmental phenotype of *chca-1* mutant worms fed HT115 (Figs 1E and S1), indicating that *chca-1*, which functions primarily in the intestine, is the gene responsible for the phenotype observed in the mutant. These results demonstrate that the copper transporter CHCA-1 is essential for the development of *C. elegans* when fed on HT115 diet.

### The developmental phenotype of *chca-1* mutant worms fed on HT115 diet results from copper deficiency

**Fig 2:**
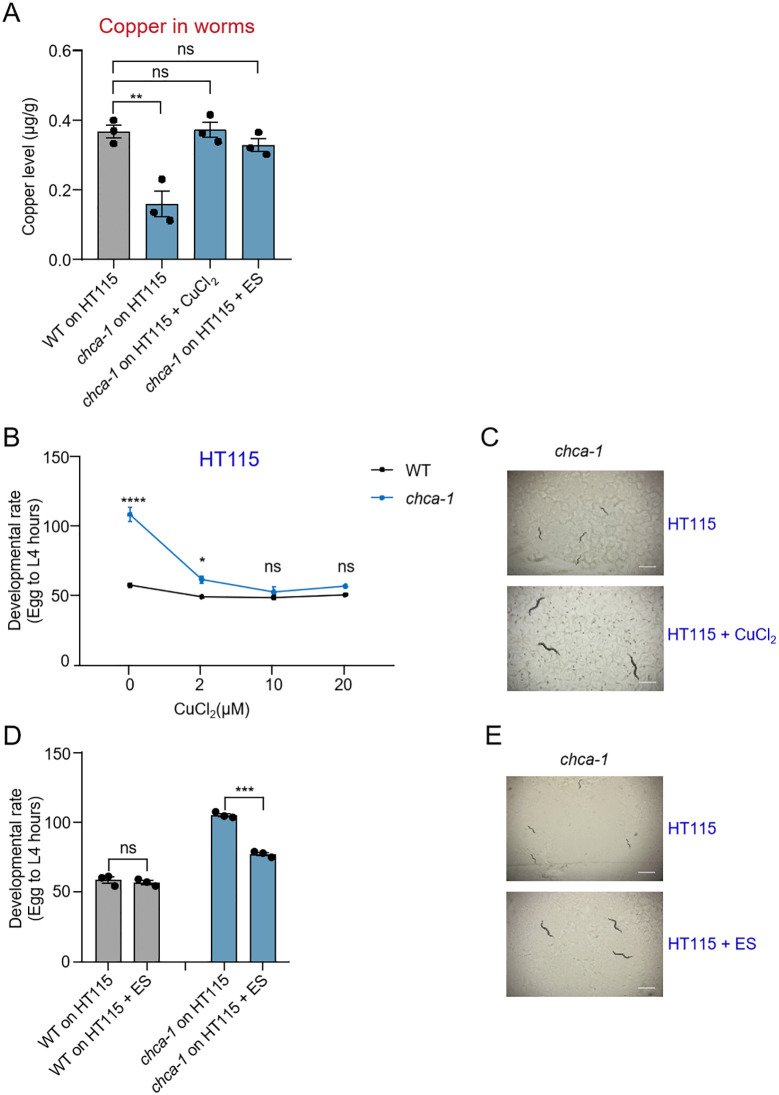
The developmental delay phenotype of *chca-1* mutant worms fed HT115 results from copper deficiency. **(A)** *chca-1(xu127)* mutant worms fed HT115 have a low copper content, which is fully rescued by supplementing CuCl_2_ (10 µM) or ES (75 µM). Data from three independent experiments (N = 3) are presented as the mean ± s.e.m. ns: no significant difference, **: P < 0.01 (one-way ANOVA with Dunnett’s test). **(B)** *chca-1(xu127)* mutant worms fed HT115 manifest a severe developmental delay defect, which is rescued by supplementing various concentrations of copper chloride (CuCl_2_). ns: no significant difference, *: P < 0.05, ****: P < 0.0001 (two-way ANOVA with Bonferroni’s test). **(C)** Representative images of *chca-1(xu127)* mutant worms fed HT115 supplemented with or without CuCl_2_ (10 µM). The images were taken 96 hours after the egg stage. Scale bar: 1 mm.**(D)** The developmental delay phenotype of *chca-1(xu127)* mutant worms fed HT115 is rescued by ES (75 µM) supplementation. ns: no significant difference, ***: P < 0.001 (two-way ANOVA with Bonferroni’s test). **(E)** Representative images of *chca-1(xu127)* mutant worms fed HT115 supplemented with or without ES (75 µM). The images were taken 96 hours after the egg stage. Scale bar: 1 mm.The developmental assay was performed at 20°C in three independent biological replicates (N = 3, n > 50). The hours needed for eggs to develop into mid L4 larvae were scored. Data are presented as the mean ± s.e.m.

As *chca-1* encodes a copper importer, we wondered if the observed developmental phenotype in *chca-1* mutant worms fed HT115 results from a deficiency in copper absorption. To address this issue, we measured the copper content in *chca-1* mutant worms fed HT115 by ICP-MS, and found that the copper concentration in mutant worms was lower than that in wild-type (Fig 2A), demonstrating copper deficiency. Importantly, the developmental phenotype of *chca-1* mutant worms fed HT115 can be fully rescued by supplementing copper (CuCl_2_) to HT115 diet (Fig 2B and 2C); furthermore, supplementing HT115 diet with the copper-binding molecule elesclomol (ES), which can facilitate the uptake of copper into the cell [26,40,41], also rescued the copper deficiency and developmental delay phenotype of *chca-1* mutant worms (Fig 2A, 2D and 2E). We further observed that *chca-1* mutant worms fed HT115 exhibited severely reduced ATP levels. This ATP deficiency was alleviated by supplementing CuCl_2_ or ES (S2 Fig). Given that the growth and development of *C. elegans* are energy-dependent, relying primarily on the mitochondrial respiratory chain (MRC) for ATP production [42], this observation suggests that copper deficiency impairs mitochondrial respiration in *chca-1* mutant worms fed HT115, thereby reducing ATP synthesis and ultimately compromising developmental progression. These data together support that the developmental phenotype in *chca-1* mutant worms fed HT115 results from copper deficiency, suggesting *chca-1* mutant worms as a promising animal model for studying copper deficiency.

### Diet switch ameliorates the developmental defect of *chca-1* mutant worms

**Fig 3:**
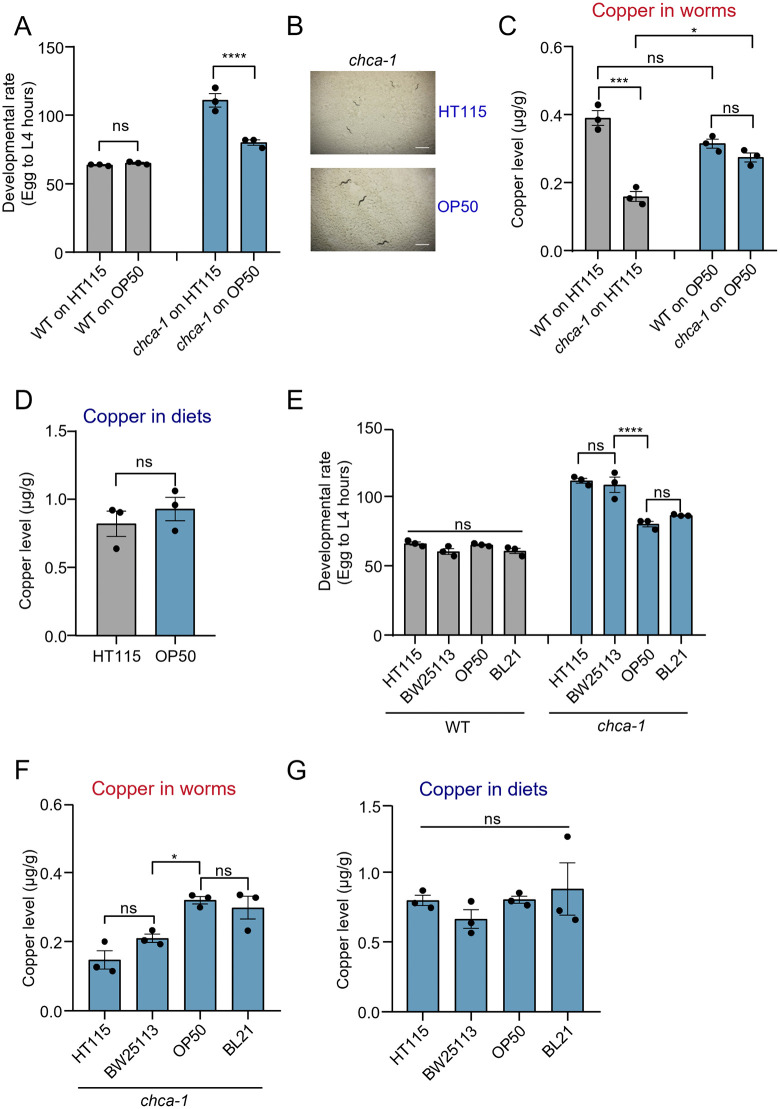
Diet switch ameliorates the developmental delay phenotype of *chca-1* mutant worms. **(A)** *chca-1(xu127)* mutant worms develop much faster on OP50 than on HT115. ns: no significant difference, ****: P < 0.0001 (two-way ANOVA with Bonferroni’s test).**(B)** Representative images of *chca-1(xu127)* mutant worms fed HT115 or OP50. The images were taken 96 hours after the egg stage. Scale bar: 1 mm.**(C)** The copper content of *chca-1(xu127)* mutant worms fed on OP50 diet is higher than that fed on HT115 diet. Data from three independent experiments (N = 3) are presented as the mean ± s.e.m. ns: no significant difference, *: P < 0.05, ***: P < 0.001 (two-way ANOVA with Tukey’s test). **(D)** The amount of copper in OP50 is similar to that in HT115. Data from three independent experiments (N = 3) are presented as the mean ± s.e.m. ns: no significant difference (t-test). **(E)** *chca-1(xu127)* mutant worms fed BW25113 exhibit a developmental delay defect similar to those fed HT115. OP50 or BL21 ameliorate the developmental delay phenotype of *chca-1(xu127)* mutant worms. ns: no significant difference, ****: P < 0.0001 (two-way ANOVA with Tukey’s test).**(F)** The copper content of *chca-1(xu127)* mutant worms fed BL21 is higher than that fed HT115 or BW25113. **(G)** The amount of copper in BL21 is similar to that in HT115 or BW25113. Data from three independent experiments (N = 3) are presented as the mean ± s.e.m. in (**F**) and **(G)**. ns: no significant difference, *: P < 0.05 (one-way ANOVA with Tukey’s test). The developmental assay was performed at 20°C in three independent biological replicates (N = 3, n > 50). The hours needed for eggs to develop into mid L4 larvae were scored. Data are presented as the mean ± s.e.m.

Surprisingly, *chca-1* mutants grew normally when fed *E. coli* OP50, another commonly used diet for *C. elegans* in the laboratory, manifesting no notable developmental deficit. A closer examination showed that the developmental rate of *chca-1* mutants fed OP50 was much faster than that of mutant worms fed HT115, though it was slightly slower than that of wild-type (Fig 3A and 3B), indicating that diet switch greatly ameliorated the developmental defect in *chca-1* mutant worms. Similarly, though slightly lower than that of wild-type worms, the copper content and ATP levels of *chca-1* mutant worms fed on OP50 diet were much higher than that fed on HT115 diet (Figs 3C and S2). This reveals a rescue effect of OP50 diet on the developmental and copper deficiency defects of *chca-1* mutant worms. Do OP50 bacteria supply more copper to the worm, thereby facilitating copper absorption and contributing to their rescue of the developmental phenotype of *chca-1* mutant worms? To our surprise, the amount of copper in OP50 was similar to that in HT115 (Fig 3D). We thus conclude that some unknown factor(s) rather than the amount of copper in OP50 diet underlies its rescue of the developmental deficit in *chca-1* mutant worms.

OP50 and HT115 were derived from two different *E. coli* bacterial lineages, B and K-12, respectively [29,43]. We thus wondered if other *E. coli* B and K-12 strains possessed a similar dietary effect on *chca-1* mutant’s developmental phenotype. To do so, we examined BW25113 bacteria (K-12 strain) [44,45], as well as BL21 bacteria (B strain) [46]. Similar to OP50 (B strain), BL21 (B strain) can also ameliorate the developmental delay phenotype of *chca-1* mutant worms (Fig 3E). Likewise, *chca-1* mutant worms fed BW25113 (K-12 strain) exhibited a severe developmental delay defect similar to those fed HT115 (K-12 strain) (Fig 3E). Consistently, the copper content of *chca-1* mutant worms fed BL21 (B strain) was higher than that fed HT115 or BW25113 (K-12 strain) (Fig 3F), even though the copper content in these four different diets was similar to each other (Fig 3G). Collectively, these results identify a differential effect of diet on *chca-1* mutant worms, suggesting that changing the diet may be an effective means to alleviate the copper deficiency as well as developmental delay phenotype of these mutant worms.

### OP50 supplementation ameliorates the developmental defect of *chca-1* mutant worms

**Fig 4:**
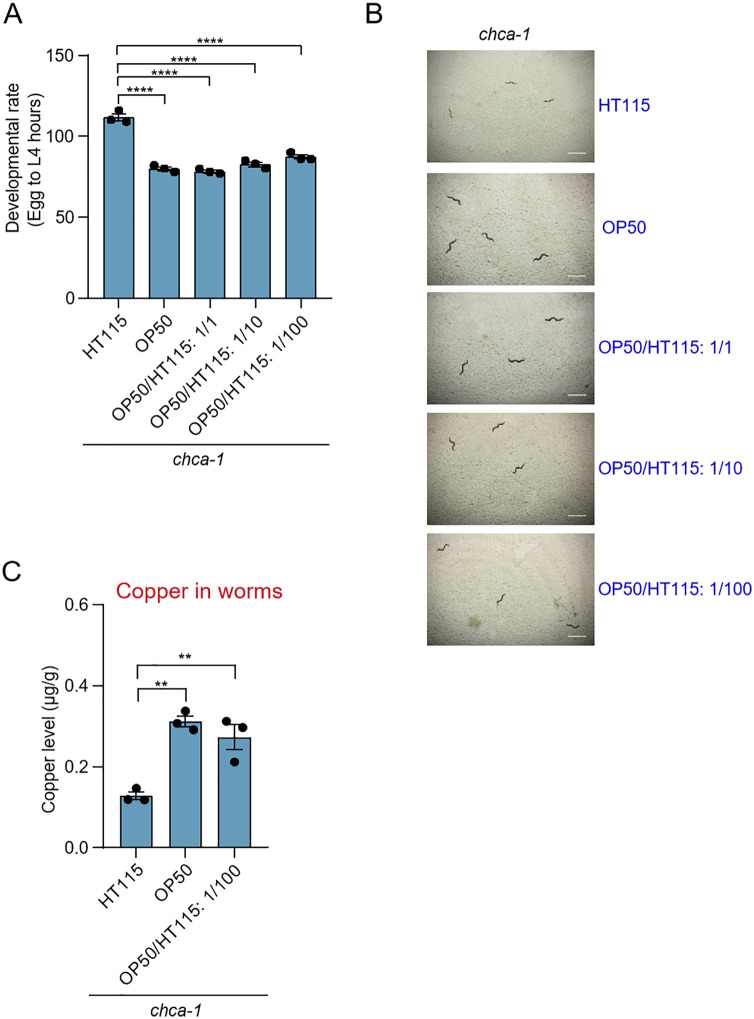
OP50 supplementation ameliorates the developmental delay phenotype of *chca-1* mutant worms. **(A)** Supplementation of 50% OP50 (OP50/HT115: 1/1), 10% OP50 (OP50/ HT115: 1/10), or 1% OP50 (OP50/HT115: 1/100) ameliorates the developmental delay phenotype of *chca-1(xu127)* mutant worms fed HT115. The developmental assay was performed at 20°C in three independent biological replicates (N = 3, n > 50). The hours needed for eggs to develop into mid L4 larvae were scored. Data are presented as the mean ± s.e.m. ****: P < 0.0001 (one-way ANOVA with Dunnett’s test).**(B)** Representative images of *chca-1(xu127)* mutant worms fed HT115 without or with OP50 supplementation. The images were taken 96 hours after the egg stage. Scale bar: 1 mm.**(C)** Supplementation with 1% OP50 (OP50/HT115: 1/100) ameliorates the copper deficiency phenotype of *chca-1(xu127)* mutant worms fed HT115. Data from three independent experiments (N = 3) are presented as the mean ± s.e.m. **: P < 0.01 (one-way ANOVA with Dunnett’s test).

We then asked whether a complete diet switch from HT115 to OP50 is required or merely supplementing OP50 to HT115 diet would be sufficient to ameliorate the developmental delay phenotype of *chca-1* mutant worms. To test this, we mixed the two bacterial diets at different ratios and examined their effect on the developmental rate of *chca-1* mutant worms. A supplement of merely 1% of OP50 to HT115 diet (OP50/HT115: 1/100) was sufficient to ameliorate the developmental delay defect of *chca-1* mutant worms (Fig 4); similarly, the copper content was also increased in mutant worms. This indicates that some component(s) in OP50 supplements played a dominant role in facilitating copper absorption, leading to the amelioration of the developmental defect of *chca-1* mutant worms even in the presence of HT115.

### GSSG supplementation ameliorates the developmental defect of *chca-1* mutant worms

**Fig 5:**
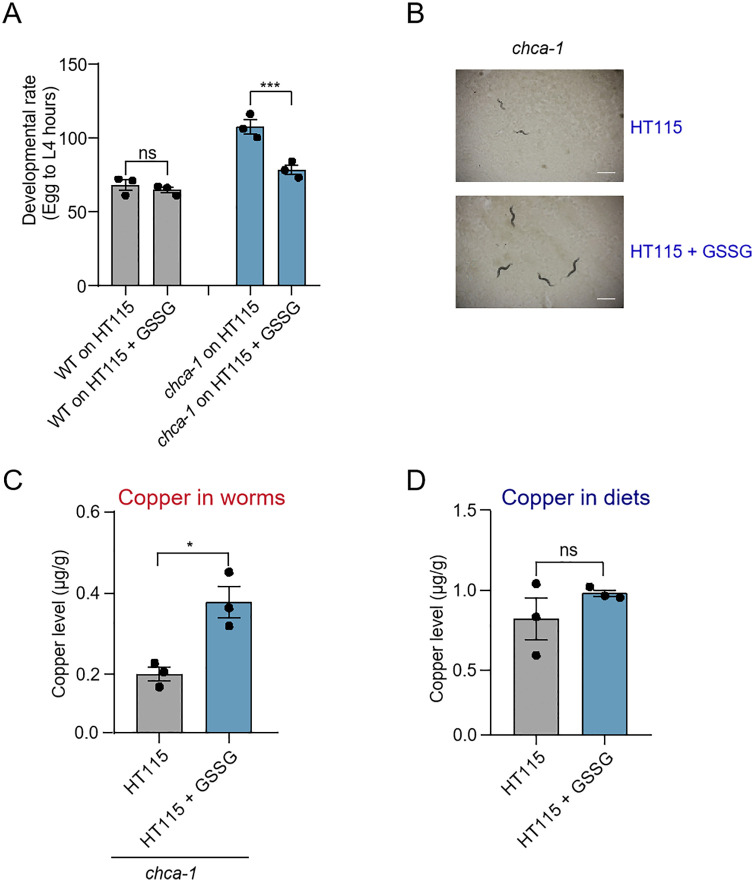
GSSG supplementation ameliorates the developmental delay phenotype of *chca-1* mutant worms. **(A)** GSSG supplementation partially rescues the developmental delay phenotype of *chca-1(xu127)* mutant worms fed HT115. GSSG (20 mM) was included in NGM plates seeded with HT115. The hours needed for eggs to develop into mid L4 larvae were scored. The developmental assay was performed at 20°C in three independent biological replicates (N = 3, n > 50). The hours needed for eggs to develop into mid L4 larvae were scored. Data are presented as the mean ± s.e.m. ns: no significant difference, ***: P < 0.001 (two-way ANOVA with Bonferroni’s test).**(B)** Representative images of *chca-1(xu127)* mutant worms fed HT115 with and without GSSG. The images were taken 96 hours after the egg stage. Scale bar: 1 mm.**(C,** **D**) GSSG supplementation elevates the copper level of *chca-1(xu127)* mutant worms fed HT115 (C), but has no significant effect on the copper level of HT115 (D). Data from three independent experiments (N = 3) are presented as the mean ± s.e.m. ns: no significant difference, *: P < 0.05 (t-test).

We then sought to identify such a component in OP50 that mediates the rescue effect of OP50 on the developmental defect in *chca-1* mutant worms fed HT115. Previous research has uncovered bacterial metabolites with higher abundance in *E. coli* B strain than that in K-12 strain [33]. We thus examined those metabolites that are > 10 times more enriched in B strain (Table 1), and explored whether supplementing such metabolites to HT115 diet ameliorates the developmental defect of *chca-1* mutant worms fed HT115. We found that a supplement of GSSG (34 times more enriched in B strain), but not other metabolites, partially rescued the developmental delay phenotype of *chca-1* mutant worms fed HT115 (Fig 5A and 5B and Table 2). Notably, metabolite set enrichment analysis revealed that the compounds listed in Table 1 are associated with glutathione metabolism (KEGG pathway map00480) (S3 Fig). Given the central role of glutathione in redox homeostasis, we asked whether GSSG supplementation might rescue copper deficiency by altering glutathione balance in the worms. However, we found that the GSH levels in *chca-1* mutants were similar when fed on HT115, OP50 or HT115 supplemented with GSSG (S4 Fig). These results indicate that GSSG supplementation does not elevate cellular GSH levels in *chca-1* mutant worms, suggesting that the observed rescue of copper deficiency is unlikely to be mediated by GSH-dependent mechanisms. To further characterize GSSG, we examined the effect of GSSG on the copper content in *chca-1* mutant worms. GSSG supplementation greatly elevated the copper level in *chca-1* mutant worms fed HT115 (Fig 5C), yet had no significant effect on the copper content of HT115 bacteria (Fig 5D). Thus, GSSG appears to primarily facilitate copper absorption in *chca-1* mutant worms to ameliorate their developmental defect rather than affect the copper amount in HT115 bacteria.

**Table 1:** Fold enrichment of metabolites in OP50 over K-12 strain.

Metabolites	Fold enrichment in OP50 over K-12 strain
Spermidine	151.7897
Norophthalmate*	42.7601
Thymidine	40.3685
Glutathione disulfide(GSSG)	34.4702
2’-Deoxyuridine	28.4348
N(1)-Acetylspermine	22.6776
2’-Deoxyguanosine	18.0391
Phosphopantetheine*	17.6487
Adenosine-2’,3’-cyclic monophosphate	13.8501
N-Carbamoylaspartate**	13.1274
2’-Deoxyinosine	12.5912
Spermine	12.0336
Nicotinate ribonucleoside*	11.7437
Ophthalmate**	10.2655

Asterisks mark the metabolites that are not commercially available (*) or preventatively costly (**) and were thus not tested. Data from (Neve et al., 2020).

**Table 2:** The effect of supplementing OP50-enriched metabolites to HT115 diet on the developmental rate of *chca-1* mutant worms.

Supplemented metabolites	Developmental rate (hours from egg to L4)	*P value* (compared to HT115 diet)
/	95.33	/
Spermidine	104.33	0.4063
Thymidine	106.33	0.3677
Glutathione disulfide (GSSG)	73.00	0.0266
2’-Deoxyuridine	91.33	0.7218
N(1)-acetylspermine	89.33	0.6045
2’-Deoxyguanosine	92.00	0.7561
Adenosine-2’,3’-cyclic monophosphate	93.67	0.8547
2’-Deoxyinosine	95.33	>0.9999
Spermine	93.67	0.8481

The working concentration of the metabolites is 20 mM or the saturation concentration of the metabolite if it is lower than 20 mM.

Severe copper deficiency can profoundly impair mitochondrial function, potentially leading to reduced ROS generation [47]. We accordingly found that mitochondrial ROS levels were reduced in *chca-1* mutant worms fed HT115 compared to wild-type worms. This pathologically low ROS level was rescued by switching to OP50 or by supplementing with CuCl_2_ or GSSG (S5A and S5B Fig). Importantly, GSSG supplementation showed no adverse effects and effectively rescued the deficits in pharyngeal pumping, brood size, and locomotion in *chca-1* mutant worms (S5C-S5E Fig), further supporting its efficacy in ameliorating copper deficiency-related phenotypes.

### *chca-1* knockin worms carrying a mutation found in a human patient exhibit a similar developmental delay phenotype

**Fig 6:**
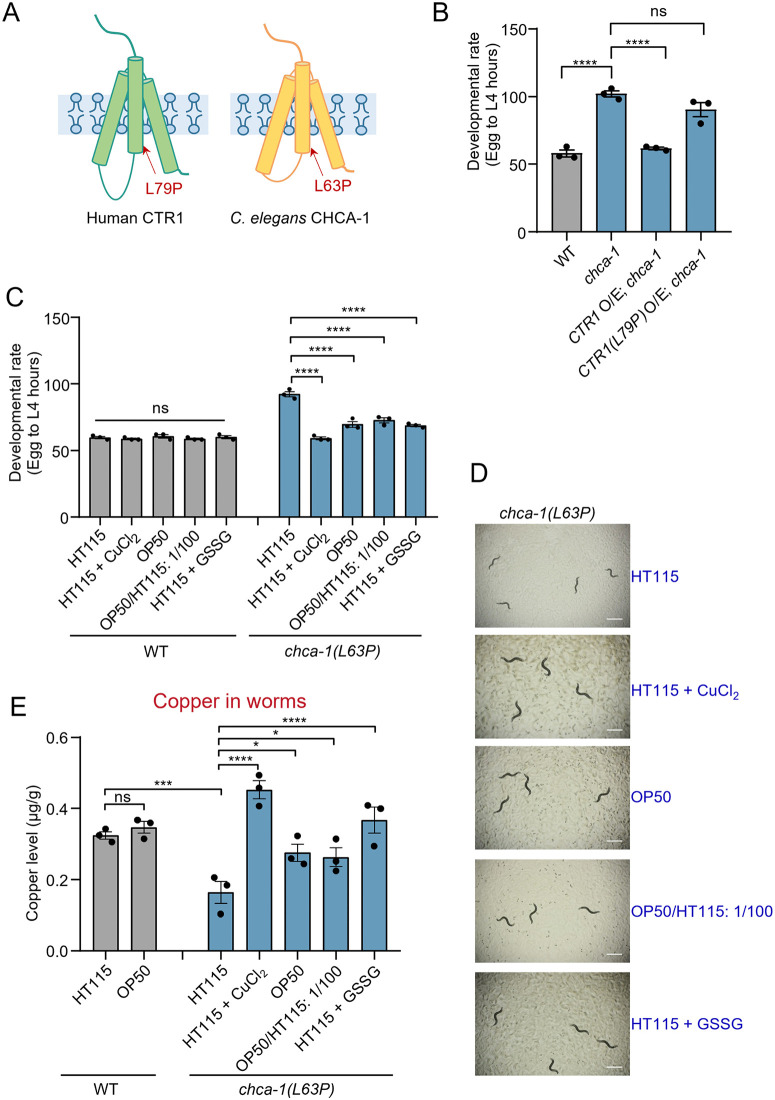
*chca-1* knockin worms carrying a mutation found in human patients exhibit a similar developmental delay phenotype (A) Schematics of worm CHCA-1 and human CTR1. The point mutation L79P in CTR1 found in a human patient diagnosed with copper deficiency disorder and the corresponding mutation L63P in worm CHCA-1 were indicated. (B) Overexpression of wild-type human CTR1 cDNA rescues the developmental delay phenotype of *chca-1(xu127)* mutant worms fed HT115, whereas overexpression of the mutant variant CTR1(L79P) fails to rescue this defect. Wild-type human CTR1 and variant CTR1(L79P) were overexpressed as transgenes under *chca-1* promoter. ns: no significant difference, ****: P < 0.0001 (one-way ANOVA with Dunnett’s test). (C) *chca-1(xu128[L63P])* knockin worms fed HT115 manifest a severe developmental delay phenotype, which is rescued by switching to OP50 diet or by supplementing HT115 diet with CuCl_2_ (10 μM), ES (75 μM), OP50 (1%), or GSSG (20 mM). The hours needed for eggs to develop into mid L4 stage larvae were scored. ns: no significant difference, ****: P < 0.0001 (two way ANOVA with Bonferroni’s test). The developmental assay in (B) and (C) was performed at 20°C in three independent biological replicates (N = 3, n > 50). The hours needed for eggs to develop into mid L4 larvae were scored. Data are presented as the mean ± s.e.m.(D) Representative images of *chca-1(xu128[L63P])* knockin worms fed OP50 and HT115 without or with CuCl_2_ (10 μM), ES (75 μM), OP50 (1%) or GSSG (20 mM) supplementation. The images were taken 96 hours after the egg stage. Scale bar: 1 mm.(E) Switching to OP50 diet, or supplementing with CuCl_2_ (10 μM), ES (75 μM), OP50 (1%) or GSSG (20 mM) increases the copper level in *chca-1(xu128[L63P])* knockin worms fed HT115. Data from three independent experiments (N = 3) are presented as the mean ± s.e.m. *: P < 0.05, ***: P < 0.001, ****: P < 0.0001 (two-way ANOVA with Tukey’s test).

*chca-1* mutant worms characterized above contained a deletion that truncates all the three putative transmembrane domains crucial for copper transporting, indicating that it is likely a null allele. On the other hand, variants of human CTR1 identified in patients all carried missense mutations [9,10]. Though such missense mutations were found in patients diagnosed with copper deficiency disorder, it is unclear whether the mutations in human CTR1 are causal for the symptoms observed in the patients. The finding that worms lacking CHCA-1 exhibited a developmental defect caused by copper deficiency offers an opportunity to address this question. L79P, a missense mutation found in human CTR1 of a patient diagnosed with copper deficiency disorder, resides in the first transmembrane domain crucial for CTR1 function [10], which is conserved in worm CHCA-1, allowing us to test this idea in worms. We expressed human CTR1 cDNA as a transgene in *chca-1* mutant background using the *chca-1* promoter, and found that the transgene fully rescued the developmental delay phenotype of *chca-1* mutant worms fed HT115 (Fig 6A and 6B). This suggests that human CTR1 can functionally substitute for worm CHCA-1 in regulating development. In contrast, L79P variant failed to rescue the developmental phenotype (Fig 6A and 6B), underscoring the functional importance of this residue. To further assess the pathological relevance of this mutation in an endogenous context, we engineered a mutant allele of *chca-1* by introducing the corresponding L63P point mutation into the endogenous locus of *chca-1* by CRISPR [48]. The resulting knockin worms, when fed on HT115, displayed a developmental delay phenotype similar to *chca-1* null mutant worms, and this phenotype can be ameliorated by switching to OP50 diet or by supplementing HT115 diet with 1% of OP50 or GSSG (Fig 6C and 6D). Similarly, the copper content in knockin worms fed on HT115 diet was lower than that in wild-type worms, and this copper deficiency phenotype can be ameliorated by switching to OP50 diet or by supplementing HT115 diet with 1% OP50 or GSSG (Fig 6E). These results demonstrate that L63P mutation in CHCA-1 recapitulated the pathogenic effect of the mutant variant of human CTR1 in the patient, suggesting that L79P mutation in human CTR1 is likely causal for copper deficiency and the pathologies observed in the patient. These data also suggest that dietary interventions, such as diet switch and supplementation, may offer a potential therapeutic approach for this severe disease.

### Dietary interventions alter the transcriptome of *chca-1* mutant worms

**Fig 7:**
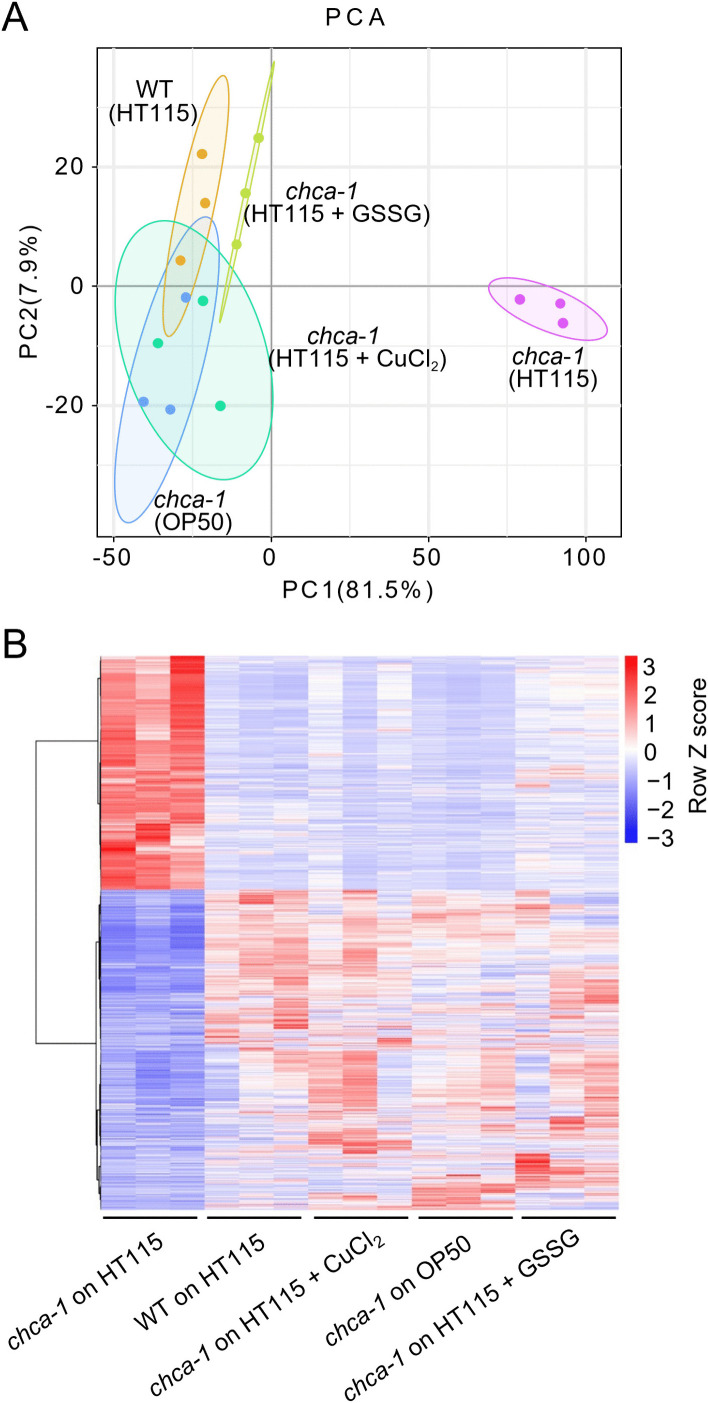
Dietary interventions reshape the transcriptome of *chca-1* mutant worms fed HT115, shifting it towards that of wild-type worms. (A) Principal components analysis (PCA) of whole-animal RNA-seq datasets shows that the transcriptome of *chca-1(xu127)* mutant worms fed HT115 clustered separately from other samples, including *chca-1(xu127)* mutants fed OP50, *chca-1(xu127)* mutants fed HT115 supplemented with GSSG, *chca-1(xu127)* mutants fed HT115 supplemented with CuCl_2_, and wild-type worms fed HT115. Three biological replicates of each condition were analyzed (N = 3).**(B)** Hierarchical clustering of RNA-seq data. For hierarchical clustering, row z scores were determined from normalized counts of differentially expressed genes (DEGs) in all samples (DESeq2, log_2_FC > 1 and P-value < 0.05). Three biological replicates of each condition were analyzed (N = 3).

To explore the mechanisms by which dietary interventions alleviate the copper deficiency and developmental delay phenotype in *chca-1* mutant worms, we profiled the transcriptome of wild-type and *chca-1* mutant worms under different dietary conditions. Principal component analysis (PCA) revealed that the transcriptome of *chca-1* mutant worms fed HT115 clustered separately from other samples, including *chca-1* mutants fed OP50, mutants fed HT115 supplemented with GSSG, and mutants fed HT115 supplemented with CuCl_2_, and wild-type worms fed HT115 (Fig 7A). This suggests that diet switch (to OP50), GSSG supplementation, and copper supplementation all altered the transcriptome of *chca-1* mutant worms (Fig 7A). Notably, the transcriptome of *chca-1* mutant worms, when switched to OP50 diet, fed HT115 supplemented with GSSG, or fed HT115 supplemented with copper, all clustered together with that of wild-type worms on the PC1 axis, which constituted the majority of PCs (81.5%). This suggests that the transcriptome of *chca-1* mutant worms under dietary interventions bears similarities to that of wild-type worms. Indeed, while the pattern of differentially regulated genes (DEGs) in *chca-1* mutant worms under dietary interventions (diet switch, GSSG supplementation and copper supplementation) was distinct from that in *chca-1* mutant worms fed HT115, it resembled that in wild-type worms (Fig 7B and S1 Table). These results demonstrate that dietary interventions remodeled the transcriptome of *chca-1* mutant worms fed HT115, shifting it towards that of wild-type worms. This also points to a potential transcriptomic mechanism underlying dietary intervention-mediated rescue of *chca-1* mutant defects.

### Dietary interventions upregulate the expression of CTR1-like copper transporters

**Fig 8:**
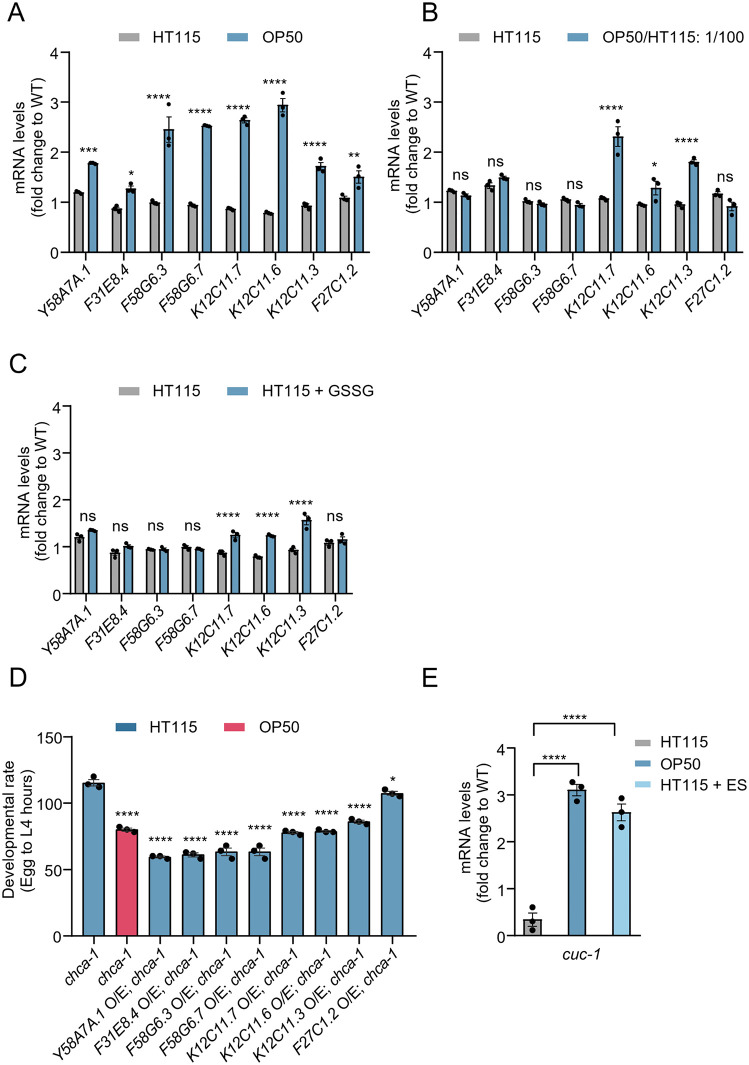
Dietary interventions upregulate the expression of CTR1-like transporters. (A) Switching the diet from HT115 to OP50 upregulated the mRNA levels of all CTR1-like transporters in *chca-1(xu127)* mutant worms. **(B, C)** 1% OP50 (**B**) or 20 mM GSSG (**C**) supplementation upregulates the mRNA level of several CTR1-like transporters in *chca-1(xu127)* mutants fed HT115. qPCR analysis of CTR1-like transporters is shown. qPCR reactions were run in triplicates for each genotype under different conditions (N = 3). Data are presented as the mean ± s.e.m. ns: no significant difference, *: P < 0.05, **: P < 0.01, ***: P < 0.001, ****: P < 0.0001 (two-way ANOVA with Bonferroni’s test).**(D)** Overexpression of CTR1-like transporters, including Y58A7A.1, F31E8.4, F58G6.3, F58G6.7, K12C11.7, K12C11.6, K12C11.3 and F27C1.2, rescues the developmental delay phenotype of *chca-1(xu127)* mutant worms fed HT115. CTR1-like transporters were overexpressed as a transgene in the intestine under *ges-1* promoter. The developmental assay was performed at 20°C in three independent biological replicates (N = 3, n > 50). The hours needed for eggs to develop into mid L4 larvae were scored. Data are presented as the mean ± s.e.m. *: P < 0.05, ****: P < 0.0001 (one-way ANOVA with Dunnett’s test).**(E)** Compared to wild-type, *chca-1(xu127)* mutants fed HT115 exhibit downregulated *cuc-1* mRNA levels, whereas switching the diet to OP50 or 75 µM ES supplementation upregulates its expression. qPCR reactions were run in triplicates for each genotype under different conditions (N = 3). Data are presented as the mean ± s.e.m. ****: P < 0.0001 (one-way ANOVA with Dunnett’s test).

Having characterized the effect of dietary interventions on *chca-1* mutant worms at the transcriptomic level, we then sought to explore the molecular mechanisms that may contribute to dietary interventions’ role in rescuing *chca-1* mutant defects. In *C. elegans*, CHCA-1 is the primary copper importer, playing a vital role in copper absorption [25]. In addition to CHCA-1, *C. elegans* genome encodes several CTR1-like proteins, including Y58A7A.1, F31E8.4, F58G6.3, F58G6.7, K12C11.7, K12C11.6, K12C11.3, F27C1.2, F01G12.1 [25]. We wondered if dietary interventions impact the expression of these CTR1-like transporters in *chca-1* mutant worms. We systemically investigated by qPCR the mRNA levels of all CTR1-like transporters in *chca-1* mutant worms under different dietary conditions with the exception of *F01G12.1*, which is a pseudogene annotated in the Wormbase. Switching the diet from HT115 to OP50 upregulated the mRNA levels of all CTR1-like transporters in *chca-1* mutant worms (Fig 8A). Supplementing OP50 or GSSG to HT115 diet also upregulated the mRNA levels of several CTR1-like transporters in *chca-1* mutants, including *K12C11.3*, *K12C11.6*, and *K12C11.7* (Fig 8B and 8C). This data suggests that dietary intervention-induced upregulation of CTR1-like transporters in *chca-1* mutant worms may contribute to the observed amelioration of *chca-1* mutant phenotype. We thus overexpressed these CTR1-like transporters as a transgene in *chca-1* mutant worms, and found that overexpression of each of these CTR1-like transporters all ameliorated the developmental defect of *chca-1* mutant worms fed HT115 (Fig 8D). Furthermore, we generated *chca-1; F58G6.3 F58G6.7* triple deletion mutant worms, and found that these worms, when fed OP50, showed no difference in development compared to *chca-1* mutant worms (S6 Fig). This result reveals a functional redundancy among CTR1-like transporters and suggests that other members, particularly the diet-sensitive genes *K12C11.3*, *K12C11.6*, and *K12C11.7*, can compensate for the loss of multiple CTR1-like genes. In addition to CTR1-like transporters, the Cu(I) chaperone gene *cuc-1*, an ATOX1 ortholog encoding a cytoplasmic Cu(I) chaperone promoting Cu(I) intracellular trafficking) [49], also exhibited a transcriptional response to copper deficiency, with expression downregulated in *chca-1* mutants fed HT115 and upregulated by OP50 feeding or ES supplementation. The elevated *cuc-1* expression in OP50- or ES-treated *chca-1* mutant worms may facilitate copper trafficking inside the cell, promoting copper homeostasis (Fig 8E). In summary, this set of data provides a potential molecular mechanism contributing to dietary intervention-induced rescue of *chca-1* mutant phenotype.

## Discussion

Due to the vital role of copper in cellular physiology, it is essential to maintain copper homeostasis in the cell for all living organisms. Various factors, such as dietary intake, age, and genetic variations in copper transporters, all influence copper homeostasis [50]. Recently, cases of severe hereditary copper deficiency disorder resulting from mutations in the copper-importing transporter CTR1 have been reported in humans [9,10]. However, no FDA-approved therapeutics are available to treat this copper deficiency disorder [51,52]. Genetic model organisms have been highly instrumental not only in understanding the basic mechanisms of human diseases but also in the development of therapeutics treating them [53–55]. Here, we developed a *C. elegans* model for copper deficiency in mutants lacking the CTR1/CHCA-1 copper transporter. *chca-1* mutant worms fed HT115 manifested a severe developmental delay defect resulting from copper deficiency, a phenotype reminiscent of that observed in human patients. A *chca-1* knockin allele, engineered to carry a point mutation found in human CTR1 of a patient diagnosed with copper deficiency disorder, manifested a phenotype similar to *chca-1* null mutant, suggesting that the mutation in human CTR1 is likely causal for the disorder in the patient. Importantly, we identified a rescue effect of dietary interventions on *chca-1* mutant phenotype, suggesting dietary interventions as a novel potential therapeutic approach for this copper deficiency disorder.

Our *C. elegans* model also serves as a system to evaluate potential therapeutics for copper deficiency disorders, including the copper ionophore elesclomol (ES). Originally developed as a chemotherapeutic agent, ES shows potential in treating copper deficiency disorders [56]. Its efficacy relies on a unique copper release mechanism where ES-bound Cu(II) must be reduced to Cu(I) by the mitochondrial reductase FDX1 before becoming bioavailable [57]. This regulated reduction enables ES to restore mitochondrial electron transport and reduce ROS levels by providing Cu(I) to superoxide dismutase (SOD). Notably, ES administration avoids inducing cuproptosis, as ES-Cu efficiently delivers copper to cytochrome c oxidase (CcO) without triggering Cu(II)-mediated toxicity [58]. Combined with FDX1-independent release pathways, this dual-mode delivery system ensures comprehensive copper distribution to both mitochondrial and non-mitochondrial cuproenzymes [57]. Our copper-deficiency *C. elegans* model further confirms the therapeutic utility of ES.

Over the past decades, multiple proteins have been identified to maintain systemic copper homeostasis, among which copper transporters play a vital role [3,59]. Copper absorption mainly depends on the copper transporter CTR1/CHCA-1, a high affinity copper importer [59]. Besides CTR1/CHCA-1, several CTR1/CHCA-1 family members have been identified in multiple species [59–63], however, their roles in copper homeostasis are largely unknown. Here, using our *C. elegans* copper deficiency model, we show that dietary interventions can upregulate the expression of CTR1/CHCA-1-like copper transporters and their overexpression can rescue the phenotype of *chca-1* mutant animals. This suggests that CTR1/CHCA-1-like transporters may play a compensatory role, whose upregulation under certain conditions such as dietary interventions, may functionally substitute for the loss of the primary copper importer CTR1/CHCA-1. While our overexpression results confirmed the functional redundancy of all eight CTR1/CHCA-1-like genes, their endogenous expression responded selectively to different dietary cues. Switching the bacterial diet from HT115 to OP50 upregulated all the tested CTR1/CHCA-1-like genes, whereas supplementation with OP50 or GSSG specifically induced a subset of genes, notably *K12C11.3*, *K12C11.6*, and *K12C11.7*. This suggests that although each family member can transport copper when overexpressed, *K12C11.3*, *K12C11.6*, and *K12C11.7* are likely more sensitive to copper status. Although it is currently unclear exactly how dietary interventions, such as diet switch and diet supplements (GSSG), upregulate the expression of CTR1/CHCA-1-like transporters, our results point to a new avenue for the development of therapeutics for copper deficiency disorder. Future efforts are needed to address this question.

The *E. coli* K-12 strain HT115 and B strain OP50 represent two most commonly used diets for feeding *C. elegans* in the laboratory. The metabolism of these two strains is quite different [33]. GSSG is one of the many metabolites enriched in B strains. Though we showed that supplementing GSSG to HT115 diet can promote copper absorption and ameliorate the developmental defect of *chca-1* mutant worms, we do not rule out the possibility that other metabolites in OP50 may possess a similar property. Indeed, the effect of supplementing GSSG is not as robust as completely switching the diet to OP50, suggesting the presence of additional components in OP50 that may contribute to its rescue effect. Indeed, metabolite profiling further identified glutathione metabolism as the most significantly enriched pathway in OP50, which includes not only GSSG but also elevated levels of polyamines such as spermidine and spermine. These metabolites serve as precursors for glutathionylspermidine and related conjugates [64,65], though their capacity to modulate copper homeostasis has yet to be determined. Identifying such additional components would facilitate the development of more effective therapeutics for this copper deficiency disorder. In addition to those copper absorption-promoting metabolites present in OP50, HT115 might produce certain metabolites that negatively interfere with copper absorption. Our current data cannot definitively distinguish between these two hypotheses regarding whether the observed rescue effect of GSSG is primarily achieved by mimicking the effect of copper absorption-promoting factors found in OP50 or by blocking those negative factors present in HT115. Future investigation will help to address this question.

Recent work has revealed an increasingly important role of diet in health and disease [66]. It is important to note that in addition to serving as a diet for *C. elegans*, bacteria are also the primary source of microbiota in the host *C. elegans* as they can inhabit in the gut of *C. elegans*, where they regulate a plethora of physiological processes of the host [67–69]. Some of those metabolites that contribute to the rescue of *chca-1* mutant phenotype may be produced by bacteria in the gut. Considering the complex interplay between microbiota and the host in the gut, our findings also raise the intriguing possibility that in addition to diet, microbiota could be a promising therapeutic target for treating copper deficiency disorder.

## Materials and methods

### Strains, genetics, and molecular biology

Wild-type: N2. *lev-1(xu100). chca-1(xu123)* (separate from *ok3201*)*. chca-1(xu127). chca-1(xu128 [L63P]). chca-1(xu127); F58G6.3 F58G6.7 deletion(xu129). Ex3951[Pchca-1::chca-1::sl2::yfp+Pmyo-2::sl2::mCherry]*; *chca-1(xu127). Ex3952[Pges-1::F58G6.3::sl2::yfp+Pmyo-2::sl2::mCherry]*; *chca-1(xu127). Ex3953[Pges-1::F58G6.7::sl2::yfp+Pmyo-2::sl2::mCherry]*; *chca-1(xu127). Ex3954[Pges-1::K12C11.3::sl2::yfp+Pmyo-2::sl2::mCherry]*; *chca-1(xu127). Ex3955[Pges-1::K12C11.6::sl2::yfp+Pmyo-2::sl2::mCherry]*; *chca-1(xu127). Ex3956[Pges-1::K12C11.7::sl2::yfp+Pmyo-2::sl2::mCherry]; chca-1(xu127). Ex3957[Pges-1::F31E8.4::sl2::yfp+Pmyo-2::sl2::mCherry]; chca-1(xu127). Ex3958[Pges-1::Y58A7A.1::sl2::yfp+Pmyo-2::sl2::mCherry]; chca-1(xu127). Ex3959[Pges-1::F27C1.2::sl2::yfp+Pmyo-2::sl2::mCherry]; chca-1(xu127). Ex3960[Pges-1::chca-1::sl2::yfp+Pmyo-2::sl2::mCherry]; chca-1(xu127). Ex3961[Pchca-1::CTR1::sl2::yfp+Pmyo-2::sl2::mCherry]; chca-1(xu127). Ex3962[Pchca-1::CTR1(L79P)::sl2::yfp+Pmyo-2::sl2::mCherry]; chca-1(xu127).*

*C. elegans* were cultivated at 20°C on nematode growth medium (NGM) plates seeded with the indicated bacteria source. Worms carrying extrachromosomal arrays were generated by injecting plasmid DNA into the hermaphrodite gonad. Human *CTR1* cDNA was amplified for functional complementation in *chca-1* deletion mutant worms. Deletion mutants were generated by CRISPR/Cas9-based genome editing using a standard protocol [36]. Knockin alleles were made by injecting RNP mixtures as described previously [48]. In brief, repair templates were amplified using unmodified primers with 100 bp homology arms. The RNP mixture was assembled at 37°C for 15 min and contained Cas9 protein, tracrRNA, and crRNA (all from IDT). crRNA corresponding DNA target sequences are 5’- AGAACGCATGAAGATAGATC-3’ (*chca-1*). The double-strand donor templates were melted right before adding them to the assembled RNPs [70]. Microinjection quality was scored using a coinjection marker, PRF4::rol-6 (su1006) plasmid. In general, 50 P0 worms were injected, singled, and maintained at 25°C for 3 days. Five plates with the highest number of F1 rollers (usually more than 20 rollers for a good injection) were selected, and 96 F1 rollers from each plate were picked and singled for further genotyping [48]. The positive insertions were confirmed by Sanger sequencing. Mutant strains were outcrossed at least six times before use.

### Bacteria diets

Fresh bacteria colonies were inoculated in Luria-Bertani (LB) medium overnight (12–14 hours) at 37°C to harvest bacteria food. The following bacterial strains were used: *E. coli* OP50 (CGC), *E. coli* HT115(DE3) (CGC), *E. coli K-12* BW25113 (JZ024 Fenghui Biotechnology), *E. coli* BL21 (JZ026 Fenghui Biotechnology).

### Developmental rate assay

The first day of adulthood was scored as day 1. For development assay, 10 day 2 adult worms (P0) were transferred to NGM plates seeded with different bacterial diets and allowed to lay eggs for 1 hour at 20°C. The time at which P0 worms were removed was set as the start time for recording the developmental process. After hatching, all larval worms were continuously monitored until the middle L4 stage. The developmental rate was quantified as the number of hours it took for eggs to develop into middle-stage L4 larvae. Three independent biological replicates were performed, with each replicate consisting of over 50 synchronized worms.

For assays involving chemical supplements, CuCl_2_ (Macklin C804816) was dissolved in aqueous solution, Elesclomol (MCE HY-12040) was dissolved in DMSO (Sigma-Aldrich D8418), and GSSG (MCE HY-D0844) was adjusted to pH = 7 with NaOH (Sigma-Aldrich S5881). Each solution was mixed with HT115 bacteria and then seeded on the NGM plates to achieve a specific final concentration.

### RB2355 outcrosses

RB2355 hermaphrodites were outcrossed into N2. *lev-1(ok3201)* deletion did not co-segregate with the developmental delay phenotype associated with RB2355. *lev-1(ok3201)* deletion was genotyped using the following primers: F primer (CGTCAATTCCACTGG) and R primer (ACGCTGCATGCACATC). The isolated mutant strain with severe developmental delay when fed HT115 was crossed with Hawaii strain CB4856 for SNP mapping; in parallel, their genomic DNA was extracted, purified, and sequenced on Novaseq 6000 sequencer (Illumina) with PE150 mode. The raw FastQ files were first filtered by fastp, discarding low-quality reads. Adapters were removed using Trimmomatic. Reads were mapped to the WBcel215 reference genome using Burrows-Wheeler Alignment Tool, and PCR duplicates were marked for filtering in the downstream analysis using Picard MarkDuplicates. Recalibration of base quality scores were then performed using GATK BaseRecalibrator. Both SNVs and Indels were called, filtered, and annotated using GATK HaplotypeCaller, GATK VariantFiltration, and ANNOVAR, respectively. The sequencing depth of the chromosomes and the variants distribution were visualized using Circos.

### ATP levels quantification

Total ATP levels were measured using the ATP Assay Kit (Beyotime, S0026) following the manufacturer’s instructions. For each replicate, 120 L4-stage worms were used. Luminescence signals were recorded using a microplate plate reader (Infinite F200 PRO, TECAN).

### Pumping rate quantification

Pharyngeal pumping was measured in 20 day 1 adult worms per group using an established method [71]. Animals were recorded for 30 seconds using a Digital Single-Lens Reflex (DSLR) camera (EOS 750D,Canon) mounted on a stereo fluorescence microscope (Discovery.V8, Zeiss). Videos in which the animal moved out of frame were excluded from analysis. Pumping rates were determined by counting pharyngeal contractions from videos replayed at 0.5 × speed.

### Brood size quantification

Brood size was assessed as previously described [72]. Briefly, for each dietary condition, one L4-stage worm was randomly selected and transferred to a fresh NGM plate seeded with the corresponding bacterial diet, with seven replicates performed per group. Worms were transferred to new plates daily until the end of egg-laying. Eggs on each plate were recorded daily, and total brood size per hermaphrodite was calculated as the sum of eggs laid over the entire reproductive period.

### Locomotion quantification

Head-thrashing locomotion was assessed in day 1 adult worms fed different diets. Individual worms were transferred to a droplet of M9 buffer on the center of a bacteria-free NGM plate. After 1 minute recovery, head thrashes were counted for 1 minute under a stereomicroscope. One thrash was defined as a complete lateral head swing from one side to the other and back [73]. 20 worms were analyzed per condition.

### GSH level quantification

GSH content was measured using a GSH assay Kit (Solarbio, BC1170). L4-stage worms were lysed by sonication, and total protein concentration was determined with a protein assay kit (Beyotime, P0397S). The luminescence was recorded with a MultiSkan SkyHigh Microplate Spectrophotometer (Thermo Fisher Scientific). Final GSH levels were normalized to total protein content for each sample and presented relative to wild-type controls.

### ROS level quantification

Reactive Oxygen Species (ROS) levels were monitored in transgenic wild-type and *chca-1* mutant worms expressing *Pvha-6::MTS::roGFP::MTS::mCherry*, a redox-sensitive probe localized to intestinal mitochondria [74]. Worms were cultured at 20°C on the indicated diets. L4-stage worms showing mCherry fluorescence were selected, immobilized with levamisole, and imaged using a confocal laser scanning microscope (FV3000, Olympus). Fluorescence intensity was quantified with Fiji/ImageJ (NIH).

### Metabolites screen

To screen for the metabolites that can rescue the developmental delay defect of *chca-1* mutant worms, nine commercially-available metabolites enriched in B strains were used, including spermidine (MCE HY-B1776), thymidine (MCE HY-N1150), oxidized glutathione (GSSG) (MCE HY-D0844), 2’-deoxyuridine(MCE HY-D0186), N(1)-acetylspermine (Sigma Aldrich-01467), 2’-deoxyguanosine (MCE HY-17563), adenosine-2’,3’-cyclic monophosphate (MCE HY-B1511), 2’-deoxyinosine (MCE HY-W008638), and spermine (MCE HY-B1777). Each metabolite was added to HT115 and seeded on NGM plates at 20 mM or their saturating concentration (final concentration: 20 mM spermidine, 6 mM thymidine, 20 mM GSSG, 20 mM 2’-deoxyuridine, 6 mM N(1)-acetylspermine, 4 mM 2’-deoxyguanosine, 6 mM adenosine-2’,3’-cyclic monophosphate, 1 mM 2’-deoxyinosine, 20 mM spermine). Synchronized eggs were seeded on the prepared plates to record developmental rate.

### Copper content measurement

The copper content of worms or bacterial diets was measured using ICP-MS as described previously [27]. For worm sample preparation, synchronized eggs were grown on NGM plates seeded with specific bacterial diet supplemented with the indicated amount of copper, GSSG, ES or OP50 until worms reached L4 stage. Worm pellets were collected and washed extensively with M9 buffer. Collected worms or bacteria were stored at -80°C before digestion. The copper content of the samples was quantified by ICP-MS (Agilent 7700). At least three independent replicates were analyzed. At least 50 mg of worms were collected for each sample. Values were normalized to wet weight of worms or bacteria.

### qRT-PCR

Total RNA was extracted from 500 synchronized L4 stage worms with TRIzol (Invitrogen). qPCR was carried out using SYBR Green (Vazyme Q711) according to the protocol provided by the manufacturer. We used *act-1* (actin) as an internal reference for normalization, and ΔΔC_t_ method was adopted to analyze qPCR data.The primers were obtained as previously described [25].

### Metabolite set enrichment analysis (MSEA)

Metabolite set enrichment analysis (MSEA) was conducted using the “Functional Enrichment” module of MetaboAnalyst 5.0. The significantly altered metabolites listed in Table 1 were included as input using KEGG identifiers, and enrichment was evaluated using the KEGG pathway library. Enrichment ratio and nominal P-values were calculated based on over-representation analysis (ORA).

### RNA sequencing and data analysis

Transcriptome analysis required ≥ 2000 synchronized worms per biological replicate. Total RNA was extracted from synchronized L4 stage worms with TRIzol (Invitrogen). Total RNA was extracted (≥ 1 μg/sample) and subjected to RNA sequencing. PCR products corresponding to 200–500 bps were enriched, quantified and sequenced on a Hiseq6000 sequencer (Illumina), and then mapped to the reference genome of *C. elegans* (WBcel235) using STRA software (version 2.5.3a) with default parameters. Reads mapped to the exon regions of each gene were counted by feature Counts (Subread-1.5.1; Bioconductor), and RPKMs were calculated.

Graphic layouts were generated using Adobe Illustrator 2022 (Adobe Systems Incorporated, USA). DESeq2 was used to analyze DEGs (log2FC > 1 and P-value < 0.05). The Principal Component Analysis (PCA) was performed using Omicshare, a free online platform for data analysis (http://www.omicshare.com/tools). The hierarchical clustering was generated using R. pheatmap. For hierarchical clustering, row z scores were determined from normalized counts of DEGs. Fold change was calculated:


$$\text{Fold}\;\text{change}\;(\text{FC})\;=\;\frac{\text{RPKMs}\;\text{of}\;\text{mutant}}{\text{RPKMs}\;\text{of}\;\text{control}}$$

### Statistics

The statistical analysis was performed using GraphPad Prism 8.0. Data values were presented as the means and standard error of means (SEM). Differences between groups were compared using Student’s t-test (two groups) or ANOVA (more than two groups) followed by recommended post hoc multiple-comparison tests. P values less than 0.05 are considered statistically significant. “N” represents the number of independent biological replicates, and “n” represents the number of worms within each biological replicate.

## Supporting information

S1 Table The z-score value of differentially expressed genes (DEGs) (related to Fig 7B). This dataset comprises the gene name, WormBase ID, log_2_(fold change), and p-value for differentially expressed genes (DEGs) identified from RNA-seq of *chca-1(xu127)* mutants and wild-type worms under various dietary conditions. Row z-scores were derived from normalized counts of these DEGs and used for hierarchical clustering in Fig 7B.
(XLSX)

S1 Fig Intestinal expression of *chca-1* rescues developmental delay in the *chca-1* mutant worms. The developmental delay of the *chca-1(xu127)* mutant worms was rescued by intestinal expression of *chca-1* wild-type gene under the control of the *ges-1* promoter. The developmental assay was performed at 20°C in three independent biological replicates (N = 3, n > 50). The hours needed for eggs to develop into mid L4 larvae were scored. Data are presented as the mean ± s.e.m. ns: no significant difference, ****: P < 0.0001 (one-way ANOVA with Dunnett’s test).
(TIF)

S2 Fig The ATP levels of wild-type and *chca-1* mutant worms fed on different conditions. The *chca-1(xu127)* mutant worms fed HT115 exhibit decreased ATP levels, which can be enhanced by switching the diet to OP50 or by supplementing HT115 with ES (75 µM) or CuCl_2_ (10 µM). In contrast, wild-type worms show no significant differences in ATP levels when fed HT115, OP50, or ES-supplemented HT115, whereas CuCl_2_ supplementation (10 µM) to HT115 resulted in a modest increase. The same number of L4 stage worms fed on different conditions were used for ATP level test. Data from three independent experiments (N = 3, n = 120) are presented as the mean ± s.e.m. ns: no significant difference, **: P < 0.01, ****: P < 0.0001 (two-way ANOVA with Bonferroni’s test).
(TIF)

S3 Fig Glutathione metabolism is the most significantly enriched pathway among metabolites enriched in OP50 versus K-12 strain. Metabolite set enrichment analysis (MSEA) of metabolites from Table 1 identifies glutathione metabolism (KEGG map00480) as the most significantly enriched pathway in OP50 compared to the K-12 strain. Dot size indicates the enrichment ratio, color represents the p-value (red, more significant), the x-axis shows −log_10_(P value), and the y-axis lists the top 8 enriched pathways.
(TIF)

S4 Fig *chca-1* mutant worms show similar GSH levels on HT115, OP50 or HT115 supplemented with GSSG. The GSH levels of *chca-1(xu127)* mutant worms fed HT115, OP50 or HT115 supplemented with 20 mM GSSG show no significant differences. The GSH contents were normalized to wild-type worms fed the corresponding diet. Data from four independent experiments (N = 4) are presented as the mean ± s.e.m. ns: no significant difference (one-way ANOVA with Dunnett’s test).
(TIF)

S5 Fig The effect of GSSG on brood size, pumping rate, locomotion and ROS levels in worms. **(A)** Representative confocal images of intestinal mitochondria in wild-type and *chca-1(xu127)* mutant worms expressing a mitochondrially targeted roGFP probe under different conditions: HT115, OP50, and HT115 supplemented with 20 mM GSSG, or HT115 supplemented with 10 µM CuCl_2_. From top to bottom: roGFP signal under 405 nm excitation (oxidized state), 488 nm excitation (reduced state), and mCherry signal under 561 nm excitation (mitochondrial morphology). Images were acquired and reconstructed using a confocal laser scanning microscope (FV3000, Olympus). Scale bar: 200 µm. **(B)** Quantification of the mitochondrial roGFP oxidation index (405/488 nm ratio) summarizing the data in (**A**). Data are presented as the mean ± s.e.m. ns: no significant difference, *: P < 0.05, ****: P < 0.0001 (two-way ANOVA with Bonferroni’s test). n = 14. **(C-E)** Supplementation of 20 mM GSSG ameliorated the defects in brood size (**C**), pumping rate (**D**), and locomotion (head thrash) (**E**) in *chca-1(xu127)* mutant worms fed HT115. Data are presented as the mean ± s.e.m. ns: no significant difference, ****: P < 0.0001 (two-way ANOVA with Bonferroni’s test). Sample sizes: n = 7 (**C**), n = 20 (**D**, **E**).
(TIF)

S6 Fig The *chca-1; F58G6.3 F58G6.7* triple deletion mutants and *chca-1* single deletion mutants exhibit similar developmental phenotype on both HT115 and OP50. Both *chca-1(xu127)* single mutants and *chca-1(xu127); F58G6.3 F58G6.7(xu129)* triple mutants fed HT115 exhibit developmental delay, which is rescued by switching to OP50 diet. No significant difference in developmental rate was observed between the triple and single mutants fed OP50. The developmental assay was performed at 20°C in three independent biological replicates (N = 3, n > 50). The hours needed for eggs to develop into mid L4 larvae were scored. Data are presented as the mean ± s.e.m. ns: no significant difference, ****: P < 0.0001 (two-way ANOVA with Bonferroni’s test).
(TIF)

S1 Data Data underlying all plots presented in this study. This dataset contains individual sheets with the data used to generate the plots included in this work.
(XLS)

## References

[1] Garza NM, Swaminathan AB, Maremanda KP, Zulkifli M, Gohil VM. Mitochondrial copper in human genetic disorders. Trends Endocrinol Metab. 2023;34(1):21–33. doi: 10.1016/j.tem.2022.11.001 36435678 PMC9780195

[2] Ge EJ, Bush AI, Casini A, Cobine PA, Cross JR, DeNicola GM, et al. Connecting copper and cancer: from transition metal signalling to metalloplasia. Nat Rev Cancer. 2022;22(2):102–13. doi: 10.1038/s41568-021-00417-2 34764459 PMC8810673

[3] Chen L, Min J, Wang F. Copper homeostasis and cuproptosis in health and disease. Signal Transduct Target Ther. 2022;7(1):378. doi: 10.1038/s41392-022-01229-y 36414625 PMC9681860

[4] Kuo YM, Zhou B, Cosco D, Gitschier J. The copper transporter CTR1 provides an essential function in mammalian embryonic development. Proc Natl Acad Sci U S A. 2001;98(12):6836–41. doi: 10.1073/pnas.111057298 11391004 PMC34439

[5] Nose Y, Kim B-E, Thiele DJ. Ctr1 drives intestinal copper absorption and is essential for growth, iron metabolism, and neonatal cardiac function. Cell Metab. 2006;4(3):235–44. doi: 10.1016/j.cmet.2006.08.009 16950140

[6] Vulpe C, Levinson B, Whitney S, Packman S, Gitschier J. Isolation of a candidate gene for Menkes disease and evidence that it encodes a copper-transporting ATPase. Nat Genet. 1993;3(1):7–13. doi: 10.1038/ng0193-7 8490659

[7] Wu J, Forbes JR, Chen HS, Cox DW. The LEC rat has a deletion in the copper transporting ATPase gene homologous to the Wilson disease gene. Nat Genet. 1994;7(4):541–5. doi: 10.1038/ng0894-541 7951327

[8] Bull PC, Thomas GR, Rommens JM, Forbes JR, Cox DW. The Wilson disease gene is a putative copper transporting P-type ATPase similar to the Menkes gene. Nat Genet. 1993;5(4):327–37. doi: 10.1038/ng1293-327 8298639

[9] Batzios S, Tal G, DiStasio AT, Peng Y, Charalambous C, Nicolaides P, et al. Newly identified disorder of copper metabolism caused by variants in CTR1, a high-affinity copper transporter. Hum Mol Genet. 2022;31(24):4121–30. doi: 10.1093/hmg/ddac156 35913762 PMC9759326

[10] Dame C, Horn D, Schomburg L, Grünhagen J, Chillon TS, Tietze A, et al. Fatal congenital copper transport defect caused by a homozygous likely pathogenic variant of SLC31A1. Clin Genet. 2023;103(5):585–9. doi: 10.1111/cge.14289 36562171

[11] Hornsten A, Lieberthal J, Fadia S, Malins R, Ha L, Xu X, et al. APL-1, a Caenorhabditis elegans protein related to the human beta-amyloid precursor protein, is essential for viability. Proc Natl Acad Sci U S A. 2007;104(6):1971–6. doi: 10.1073/pnas.0603997104 17267616 PMC1794273

[12] Levitan D, Doyle TG, Brousseau D, Lee MK, Thinakaran G, Slunt HH, et al. Assessment of normal and mutant human presenilin function in Caenorhabditis elegans. Proc Natl Acad Sci U S A. 1996;93(25):14940–4. doi: 10.1073/pnas.93.25.14940 8962160 PMC26241

[13] Levitan D, Greenwald I. Facilitation of lin-12-mediated signalling by sel-12, a Caenorhabditis elegans S182 Alzheimer’s disease gene. Nature. 1995;377(6547):351–4. doi: 10.1038/377351a0 7566091

[14] Wittenburg N, Eimer S, Lakowski B, Röhrig S, Rudolph C, Baumeister R. Presenilin is required for proper morphology and function of neurons in C. elegans. Nature. 2000;406(6793):306–9. doi: 10.1038/35018575 10917532

[15] Rani N, Alam MM, Jamal A, Bin Ghaffar U, Parvez S. Caenorhabditis elegans: A transgenic model for studying age-associated neurodegenerative diseases. Ageing Res Rev. 2023;91:102036. doi: 10.1016/j.arr.2023.102036 37598759

[16] Barr MM, DeModena J, Braun D, Nguyen CQ, Hall DH, Sternberg PW. The Caenorhabditis elegans autosomal dominant polycystic kidney disease gene homologs lov-1 and pkd-2 act in the same pathway. Curr Biol. 2001;11(17):1341–6. doi: 10.1016/s0960-9822(01)00423-7 11553327

[17] Barr MM, Sternberg PW. A polycystic kidney-disease gene homologue required for male mating behaviour in C. elegans. Nature. 1999;401(6751):386–9. doi: 10.1038/43913 10517638

[18] Delmas P. Polycystins: from mechanosensation to gene regulation. Cell. 2004;118(2):145–8. doi: 10.1016/j.cell.2004.07.007 15260985

[19] Ogg S, Paradis S, Gottlieb S, Patterson GI, Lee L, Tissenbaum HA, et al. The Fork head transcription factor DAF-16 transduces insulin-like metabolic and longevity signals in C. elegans. Nature. 1997;389(6654):994–9. doi: 10.1038/40194 9353126

[20] Shen P, Yue Y, Park Y. A living model for obesity and aging research: Caenorhabditis elegans. Crit Rev Food Sci Nutr. 2018;58(5):741–54. doi: 10.1080/10408398.2016.1220914 27575804

[21] Shen P, Yue Y, Zheng J, Park Y. Caenorhabditis elegans: A Convenient In Vivo Model for Assessing the Impact of Food Bioactive Compounds on Obesity, Aging, and Alzheimer’s Disease. Annu Rev Food Sci Technol. 2018;9:1–22. doi: 10.1146/annurev-food-030117-012709 29261338

[22] Sternberg PW, Han M. Genetics of RAS signaling in C. elegans. Trends Genet. 1998;14(11):466–72. doi: 10.1016/s0168-9525(98)01592-3 9825675

[23] Kaletta T, Hengartner MO. Finding function in novel targets: C. elegans as a model organism. Nat Rev Drug Discov. 2006;5(5):387–98. doi: 10.1038/nrd2031 16672925

[24] Chun H, Sharma AK, Lee J, Chan J, Jia S, Kim B-E. The Intestinal Copper Exporter CUA-1 Is Required for Systemic Copper Homeostasis in Caenorhabditis elegans. J Biol Chem. 2017;292(1):1–14. doi: 10.1074/jbc.M116.760876 27881675 PMC5217669

[25] Yuan S, Sharma AK, Richart A, Lee J, Kim B-E. CHCA-1 is a copper-regulated CTR1 homolog required for normal development, copper accumulation, and copper-sensing behavior in Caenorhabditis elegans. J Biol Chem. 2018;293(28):10911–25. doi: 10.1074/jbc.RA118.003503 29784876 PMC6052217

[26] Yuan S, Korolnek T, Kim B-E. Oral Elesclomol Treatment Alleviates Copper Deficiency in Animal Models. Front Cell Dev Biol. 2022;10:856300. doi: 10.3389/fcell.2022.856300 35433682 PMC9010564

[27] Lee J, Prohaska JR, Thiele DJ. Essential role for mammalian copper transporter Ctr1 in copper homeostasis and embryonic development. Proc Natl Acad Sci U S A. 2001;98(12):6842–7. doi: 10.1073/pnas.111058698 11391005 PMC34440

[28] Xiao R, Chun L, Ronan EA, Friedman DI, Liu J, Xu XZS. RNAi Interrogation of Dietary Modulation of Development, Metabolism, Behavior, and Aging in C. elegans. Cell Rep. 2015;11(7):1123–33. doi: 10.1016/j.celrep.2015.04.024 25959815 PMC4439342

[29] Timmons L, Court DL, Fire A. Ingestion of bacterially expressed dsRNAs can produce specific and potent genetic interference in Caenorhabditis elegans. Gene. 2001;263(1–2):103–12. doi: 10.1016/s0378-1119(00)00579-5 11223248

[30] Gracida X, Eckmann CR. Fertility and germline stem cell maintenance under different diets requires nhr-114/HNF4 in C. elegans. Curr Biol. 2013;23(7):607–13. doi: 10.1016/j.cub.2013.02.034 23499532

[31] Pang S, Curran SP. Adaptive capacity to bacterial diet modulates aging in C. elegans. Cell Metab. 2014;19(2):221–31. doi: 10.1016/j.cmet.2013.12.005 24440036 PMC3979424

[32] Verma S, Jagtap U, Goyala A, Mukhopadhyay A. A novel gene-diet pair modulates C. elegans aging. PLoS Genet. 2018;14(8):e1007608. doi: 10.1371/journal.pgen.1007608 30125273 PMC6117094

[33] Neve IAA, Sowa JN, Lin C-CJ, Sivaramakrishnan P, Herman C, Ye Y, et al. Escherichia coli Metabolite Profiling Leads to the Development of an RNA Interference Strain for Caenorhabditis elegans. G3 (Bethesda). 2020;10(1):189–98. doi: 10.1534/g3.119.400741 31712257 PMC6945014

[34] Zhou J-J, Chun L, Liu J-F. A Comprehensive Understanding of Dietary Effects on C. elegans Physiology. Curr Med Sci. 2019;39(5):679–84. doi: 10.1007/s11596-019-2091-6 31612382

[35] Albuquerque EX, Pereira EFR, Alkondon M, Rogers SW. Mammalian nicotinic acetylcholine receptors: from structure to function. Physiol Rev. 2009;89(1):73–120. doi: 10.1152/physrev.00015.2008 19126755 PMC2713585

[36] Dickinson DJ, Ward JD, Reiner DJ, Goldstein B. Engineering the Caenorhabditis elegans genome using Cas9-triggered homologous recombination. Nat Methods. 2013;10(10):1028–34. doi: 10.1038/nmeth.2641 23995389 PMC3905680

[37] Davis MW, Hammarlund M, Harrach T, Hullett P, Olsen S, Jorgensen EM. Rapid single nucleotide polymorphism mapping in C. elegans. BMC Genomics. 2005;6:118. doi: 10.1186/1471-2164-6-118 16156901 PMC1242227

[38] Sarin S, Prabhu S, O’Meara MM, Pe’er I, Hobert O. Caenorhabditis elegans mutant allele identification by whole-genome sequencing. Nat Methods. 2008;5(10):865–7. doi: 10.1038/nmeth.1249 18677319 PMC2574580

[39] Ren F, Logeman BL, Zhang X, Liu Y, Thiele DJ, Yuan P. X-ray structures of the high-affinity copper transporter Ctr1. Nat Commun. 2019;10(1):1386. doi: 10.1038/s41467-019-09376-7 30918258 PMC6437178

[40] Guthrie LM, Soma S, Yuan S, Silva A, Zulkifli M, Snavely TC, et al. Elesclomol alleviates Menkes pathology and mortality by escorting Cu to cuproenzymes in mice. Science. 2020;368(6491):620–5. doi: 10.1126/science.aaz8899 32381719 PMC7304446

[41] Soma S, Latimer AJ, Chun H, Vicary AC, Timbalia SA, Boulet A, et al. Elesclomol restores mitochondrial function in genetic models of copper deficiency. Proc Natl Acad Sci U S A. 2018;115(32):8161–6. doi: 10.1073/pnas.1806296115 30038027 PMC6094114

[42] Tsang WY, Sayles LC, Grad LI, Pilgrim DB, Lemire BD. Mitochondrial respiratory chain deficiency in Caenorhabditis elegans results in developmental arrest and increased life span. J Biol Chem. 2001;276(34):32240–6. doi: 10.1074/jbc.M103999200 11410594

[43] Brenner S. The genetics of Caenorhabditis elegans. Genetics. 1974;77(1):71–94. doi: 10.1093/genetics/77.1.71 4366476 PMC1213120

[44] Baba T, Ara T, Hasegawa M, Takai Y, Okumura Y, Baba M, et al. Construction of Escherichia coli K-12 in-frame, single-gene knockout mutants: the Keio collection. Mol Syst Biol. 2006;2:2006.0008. doi: 10.1038/msb4100050 16738554 PMC1681482

[45] Blattner FR, Plunkett G 3rd, Bloch CA, Perna NT, Burland V, Riley M, et al. The complete genome sequence of Escherichia coli K-12. Science. 1997;277(5331):1453–62. doi: 10.1126/science.277.5331.1453 9278503

[46] Kim S, Jeong H, Kim E-Y, Kim JF, Lee SY, Yoon SH. Genomic and transcriptomic landscape of Escherichia coli BL21(DE3). Nucleic Acids Res. 2017;45(9):5285–93. doi: 10.1093/nar/gkx228 28379538 PMC5435950

[47] Bustos RI, Jensen EL, Ruiz LM, Rivera S, Ruiz S, Simon F, et al. Copper deficiency alters cell bioenergetics and induces mitochondrial fusion through up-regulation of MFN2 and OPA1 in erythropoietic cells. Biochem Biophys Res Commun. 2013;437(3):426–32. doi: 10.1016/j.bbrc.2013.06.095 23831624

[48] Ghanta KS, Ishidate T, Mello CC. Microinjection for precision genome editing in Caenorhabditis elegans. STAR Protoc. 2021;2(3):100748. doi: 10.1016/j.xpro.2021.100748 34505086 PMC8417391

[49] Wakabayashi T, Nakamura N, Sambongi Y, Wada Y, Oka T, Futai M. Identification of the copper chaperone, CUC-1, in Caenorhabditis elegans: tissue specific co-expression with the copper transporting ATPase, CUA-1. FEBS Lett. 1998;440(1–2):141–6. doi: 10.1016/s0014-5793(98)01431-8 9862443

[50] Prohaska JR. Impact of copper deficiency in humans. Ann N Y Acad Sci. 2014;1314:1–5. doi: 10.1111/nyas.12354 24517364

[51] Kaler SG, Holmes CS, Goldstein DS, Tang J, Godwin SC, Donsante A, et al. Neonatal diagnosis and treatment of Menkes disease. N Engl J Med. 2008;358(6):605–14. doi: 10.1056/NEJMoa070613 18256395 PMC3477514

[52] Kaler SG. ATP7A-related copper transport diseases-emerging concepts and future trends. Nat Rev Neurol. 2011;7(1):15–29. doi: 10.1038/nrneurol.2010.180 21221114 PMC4214867

[53] Bergers G, Javaherian K, Lo KM, Folkman J, Hanahan D. Effects of angiogenesis inhibitors on multistage carcinogenesis in mice. Science. 1999;284(5415):808–12. doi: 10.1126/science.284.5415.808 10221914

[54] Huss WJ, Barrios RJ, Greenberg NM. SU5416 selectively impairs angiogenesis to induce prostate cancer-specific apoptosis. Mol Cancer Ther. 2003;2(7):611–6. 12883033

[55] Gitler AD, Lehmann R. Modeling human disease. Science. 2012;337(6092):269. doi: 10.1126/science.1227179 22822114

[56] Zheng P, Zhou C, Lu L, Liu B, Ding Y. Elesclomol: a copper ionophore targeting mitochondrial metabolism for cancer therapy. J Exp Clin Cancer Res. 2022;41(1):271. doi: 10.1186/s13046-022-02485-0 36089608 PMC9465867

[57] Zulkifli M, Spelbring AN, Zhang Y, Soma S, Chen S, Li L, et al. FDX1-dependent and independent mechanisms of elesclomol-mediated intracellular copper delivery. Proc Natl Acad Sci U S A. 2023;120(10):e2216722120. doi: 10.1073/pnas.2216722120 36848556 PMC10013847

[58] Zulkifli M, Maremanda KP, Okonkwo AU, Farid I, Gohil VM. Elesclomol rescues mitochondrial copper deficiency in disease models without triggering cuproptosis. J Pharmacol Exp Ther. 2025;392(2):100048. doi: 10.1016/j.jpet.2024.100048 40023603 PMC12718110

[59] Ohrvik H, Thiele DJ. How copper traverses cellular membranes through the mammalian copper transporter 1, Ctr1. Ann N Y Acad Sci. 2014;1314:32–41. doi: 10.1111/nyas.12371 24697869 PMC4158275

[60] Dancis A, Yuan DS, Haile D, Askwith C, Eide D, Moehle C, et al. Molecular characterization of a copper transport protein in S. cerevisiae: an unexpected role for copper in iron transport. Cell. 1994;76(2):393–402. doi: 10.1016/0092-8674(94)90345-x 8293472

[61] Knight SA, Labbé S, Kwon LF, Kosman DJ, Thiele DJ. A widespread transposable element masks expression of a yeast copper transport gene. Genes Dev. 1996;10(15):1917–29. doi: 10.1101/gad.10.15.1917 8756349

[62] Zhou H, Thiele DJ. Identification of a novel high affinity copper transport complex in the fission yeast Schizosaccharomyces pombe. J Biol Chem. 2001;276(23):20529–35. doi: 10.1074/jbc.M102004200 11274192

[63] Turski ML, Thiele DJ. Drosophila Ctr1A functions as a copper transporter essential for development. J Biol Chem. 2007;282(33):24017–26. doi: 10.1074/jbc.M703792200 17573340

[64] Dubin DT. Evidence for conjugates between polyamines and glutathione in E. coli. Biochemical and Biophysical Research Communications. 1959;1(5):262–5. doi: 10.1016/0006-291x(59)90034-8

[65] Chattopadhyay MK, Chen W, Tabor H. Escherichia coli glutathionylspermidine synthetase/amidase: phylogeny and effect on regulation of gene expression. FEMS Microbiol Lett. 2013;338(2):132–40. doi: 10.1111/1574-6968.12035 23106382 PMC3812799

[66] Xiao Y-L, Gong Y, Qi Y-J, Shao Z-M, Jiang Y-Z. Effects of dietary intervention on human diseases: molecular mechanisms and therapeutic potential. Signal Transduct Target Ther. 2024;9(1):59. doi: 10.1038/s41392-024-01771-x 38462638 PMC10925609

[67] Lee Y-U, Fox BW, Guo R, Curtis BJ, Yu J, Kim S, et al. Host-microbe interactions rewire metabolism in a C. elegans model of leucine breakdown deficiency. Nat Metab. 2024;6(8):1584–600. doi: 10.1038/s42255-024-01098-5 39117959 PMC11670331

[68] Scott TA, Quintaneiro LM, Norvaisas P, Lui PP, Wilson MP, Leung KY, et al. Host-Microbe Co-metabolism Dictates Cancer Drug Efficacy in *C. elegans*. Cell. 2017;169(3):442–56.e18.doi: 10.1016/j.cell.2017.03.040 28431245 PMC5406385

[69] Han B, Sivaramakrishnan P, Lin C-CJ, Neve IAA, He J, Tay LWR, et al. Microbial Genetic Composition Tunes Host Longevity. Cell. 2017;169(7):1249-1262.e13. doi: 10.1016/j.cell.2017.05.036 28622510 PMC5635830

[70] Ghanta KS, Mello CC. Melting dsDNA Donor Molecules Greatly Improves Precision Genome Editing in Caenorhabditis elegans. Genetics. 2020;216(3):643–50. doi: 10.1534/genetics.120.303564 32963112 PMC7648581

[71] Croft JC, Colunga A, Solh L, Dillon MK, Lee TW-S. Pharyngeal pumping rate does not reflect lifespan extension in C. elegans transgenerational longevity mutants. MicroPubl Biol. 2023;2023:10.17912/micropub.biology.000719. doi: 10.17912/micropub.biology.000719 36793895 PMC9923420

[72] Yu L, Yan X, Ye C, Zhao H, Chen X, Hu F, et al. Bacterial Respiration and Growth Rates Affect the Feeding Preferences, Brood Size and Lifespan of Caenorhabditis elegans. PLoS One. 2015;10(7):e0134401. doi: 10.1371/journal.pone.0134401 26222828 PMC4519269

[73] Cao X, Wang X, Chen H, Li H, Tariq M, Wang C, et al. Neurotoxicity of nonylphenol exposure on Caenorhabditis elegans induced by reactive oxidative species and disturbance synthesis of serotonin. Environ Pollut. 2019;244:947–57. doi: 10.1016/j.envpol.2018.09.140 30469289

[74] Labuschagne CF, Brenkman AB. Current methods in quantifying ROS and oxidative damage in Caenorhabditis elegans and other model organism of aging. Ageing Res Rev. 2013;12(4):918–30. doi: 10.1016/j.arr.2013.09.003 24080227

